# Structure of a bacterial ATP synthase

**DOI:** 10.7554/eLife.43128

**Published:** 2019-02-06

**Authors:** Hui Guo, Toshiharu Suzuki, John L Rubinstein

**Affiliations:** 1 The Hospital for Sick Children Research InstituteTorontoCanada; 2 Department of Medical BiophysicsThe University of TorontoTorontoCanada; 3 Laboratory for Chemistry and Life Science, Institute of Innovative ResearchTokyo Institute of TechnologyYokohamaJapan; 4 Department of Molecular BioscienceKyoto-Sangyo UniversityKyotoJapan; 5 Department of BiochemistryThe University of TorontoTorontoCanada; University of California, BerkeleyUnited States; University of OxfordUnited Kingdom

**Keywords:** ATP synthase, Bacillus PS3, membrane, protein, structure, cryo-EM, Other

## Abstract

ATP synthases produce ATP from ADP and inorganic phosphate with energy from a transmembrane proton motive force. Bacterial ATP synthases have been studied extensively because they are the simplest form of the enzyme and because of the relative ease of genetic manipulation of these complexes. We expressed the *Bacillus* PS3 ATP synthase in *Eschericia coli*, purified it, and imaged it by cryo-EM, allowing us to build atomic models of the complex in three rotational states. The position of subunit *ε* shows how it is able to inhibit ATP hydrolysis while allowing ATP synthesis. The architecture of the membrane region shows how the simple bacterial ATP synthase is able to perform the same core functions as the equivalent, but more complicated, mitochondrial complex. The structures reveal the path of transmembrane proton translocation and provide a model for understanding decades of biochemical analysis interrogating the roles of specific residues in the enzyme.

## Introduction

Adenosine triphosphate (ATP) synthases are multi-subunit protein complexes that use an electrochemical proton motive force across a membrane to make the cell’s supply of ATP from adenosine diphosphate (ADP) and inorganic phosphate (Pi). These enzymes are found in bacteria and chloroplasts as monomers, and in mitochondria as rows of dimers that bend the inner membrane to facilitate formation of the mitochondrial cristae (Davies et al., 2012; Paumard et al., 2002). Proton translocation across the membrane-embedded F_O_ region of the complex occurs via two offset half-channels (Vik and Antonio, 1994; Junge et al., 1997). Studies with *Bacillus* PS3 ATP synthase in liposomes showed that proton translocation may be driven by ΔpH or ΔΨ alone (Soga et al., 2012). The passage of protons causes rotation of a rotor subcomplex, inducing conformational change in the catalytic F_1_ region to produce ATP (Walker, 2013) while a peripheral stalk subcomplex holds the F_1_ region stationary relative to the spinning rotor during catalysis. For the mitochondrial enzyme, X-ray crystallography has been used to determine structures of the soluble F_1_ region (Abrahams et al., 1994), partial structures of the peripheral stalk subcomplex alone (Dickson et al., 2006) and with the F_1_ region (Rees et al., 2009), and structures of the F_1_ region with the membrane-embedded ring of *c*-subunits attached (Stock et al., 1999; Watt et al., 2010). Recent breakthroughs in electron cryomicroscopy (cryo-EM) allowed the structures of the membrane-embedded F_O_ regions from mitochondrial and chloroplast ATP synthases to be determined to near-atomic resolutions (Guo et al., 2017; Klusch et al., 2017; Srivastava et al., 2018; Hahn et al., 2018).

Compared to their mitochondrial counterparts, bacterial ATP synthases have a simpler subunit composition. The F_1_ region consists of subunits *α_3_β_3_γδε*, while the F_O_ region is usually formed by three subunits with the stoichiometry *ab_2_c_9-15_*. Chloroplasts and a few bacteria, such as *Paracoccus denitrificans*, possess two different but homologous copies of subunit *b*, named subunits *b* and *b’* (Walker, 2013). Each copy of subunit α and *β* contains a nucleotide binding site. The non-catalytic *α* subunits each bind to a magnesium ion (Mg^2+^) and a nucleotide, while the catalytic *β* subunits can adopt different conformations and bind to Mg-ADP (*β_DP_*), Mg-ATP (*β_TP_*), or remain empty (*β_E_*). Crystal structures of bacterial F_1_-ATPases and *c*-rings from the F_O_ regions of several species have been determined (Stocker et al., 2007; Cingolani and Duncan, 2011; Morales-Rios et al., 2015; Shirakihara et al., 2015; Ferguson et al., 2016; Pogoryelov et al., 2009; Preiss et al., 2010; Preiss et al., 2013; Preiss et al., 2015). Structures of intact ATP synthases from *E. coli* have been determined to overall resolutions of 6 to 7 Å by cryo-EM, with the F_O_ region showing lower quality than the rest of the maps, presumably due to conformational flexibility (Sobti et al., 2016). In structures of both intact ATP synthase (Sobti et al., 2016) and dissociated F_1_-ATPase (Cingolani and Duncan, 2011; Shirakihara et al., 2015) from bacteria, subunit *ε* adopts an ‘up’ conformation that inhibits the ATP hydrolysis by the enzyme. In the thermophilic bacterium *Bacillus* PS3, this subunit *ε* mediated inhibition is dependent on the concentration of free ATP (Kato et al., 1997; Suzuki et al., 2003; Saita et al., 2010). Low ATP concentrations (e.g. <0.7 mM) promote the inhibitory *up* conformation while a permissive ‘down’ conformation can be induced by a high concentration of ATP (e.g. >1 mM). This mechanism would allow the *Bacillus* PS3 ATP synthase to run in reverse, establishing a proton motive force by ATP hydrolysis, when the ATP concentration is sufficient to do so without depleting the cell’s supply of ATP. In *E. coli*, however, in the absence of a sufficient proton motive force to drive ATP synthesis, inhibition of ATP hydrolysis by subunit *ε* persists even when the concentration of free ATP is high (Laget and Smith, 1979; Sekiya et al., 2010).

**Figure 1.**
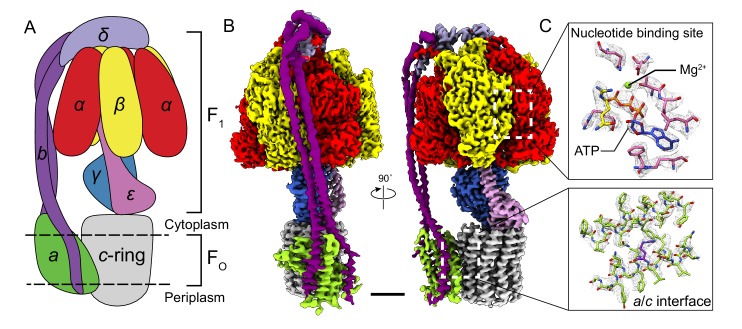
Overall structure of *Bacillus* PS3 ATP synthase. (**A**) Cartoon of ATP synthase. (**B**) Cryo-EM map of ATP synthase with subunits coloured the same as the cartoon. (**C**) Example map density that allowed construction of an atomic model. Scale bar, 30 Å.

Although bacterial ATP synthases have been subjected to extensive biochemical analysis, high-resolution structural information is lacking for the intact enzyme or the membrane-embedded proton-conducting subunit *a* and the associated subunit *b*. We determined structures of intact ATP synthase from *Bacillus* PS3 in three rotational states by cryo-EM. The structures reached overall resolutions of 3.0, 3.0, and 3.2 Å (Figure 1), allowing construction of nearly complete atomic models for the entire complex. The structures reveal how loops in subunit *a* of the bacterial enzyme fill the role of additional subunits in the F_O_ region of the mitochondrial enzyme. Most significantly, the structures provide a framework for understanding decades of mutagenesis experiments designed to probe the mechanism of ATP synthases.

**Figure 1—figure supplement 1.**
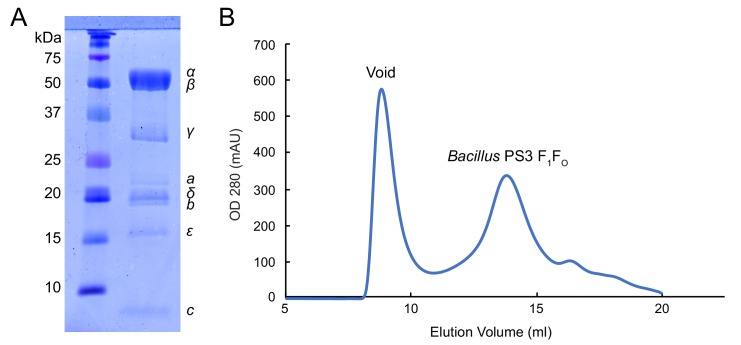
*Bacillus* PS3 ATP synthase purification. (**A**) SDS-PAGE of GDN solubilized *Bacillus* PS3 ATP synthase. (**B**) Size-exclusion chromatography of *Bacillus* PS3 ATP synthase.

**Figure 1—figure supplement 2.**
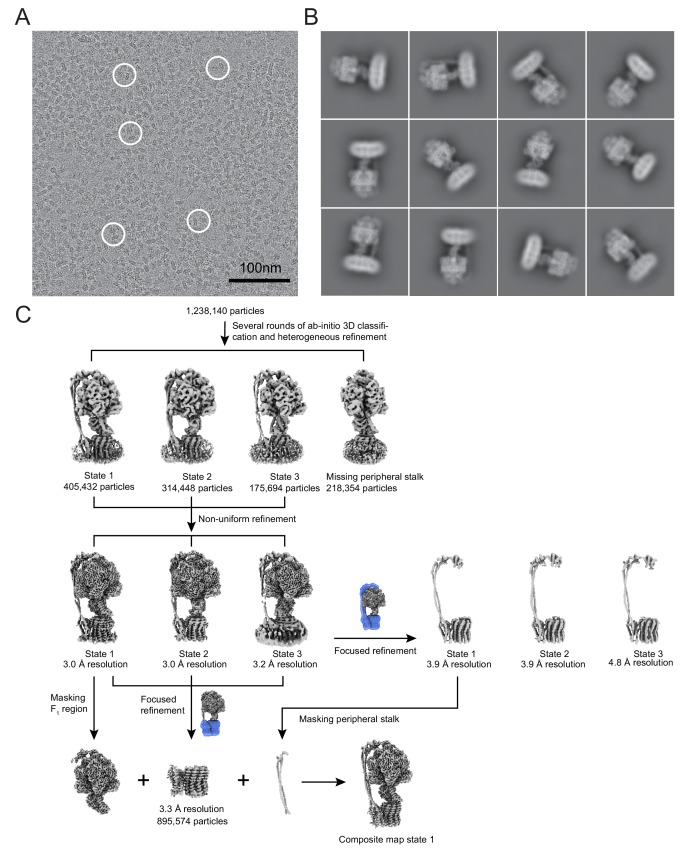
Cryo-EM image processing. (**A**) Example micrograph of *Bacillus* PS3 ATP synthase. (**B**) Representative 2D class averages of *Bacillus* PS3 ATP synthase. (**C**) Image processing workflow.

**Figure 1—figure supplement 3.**
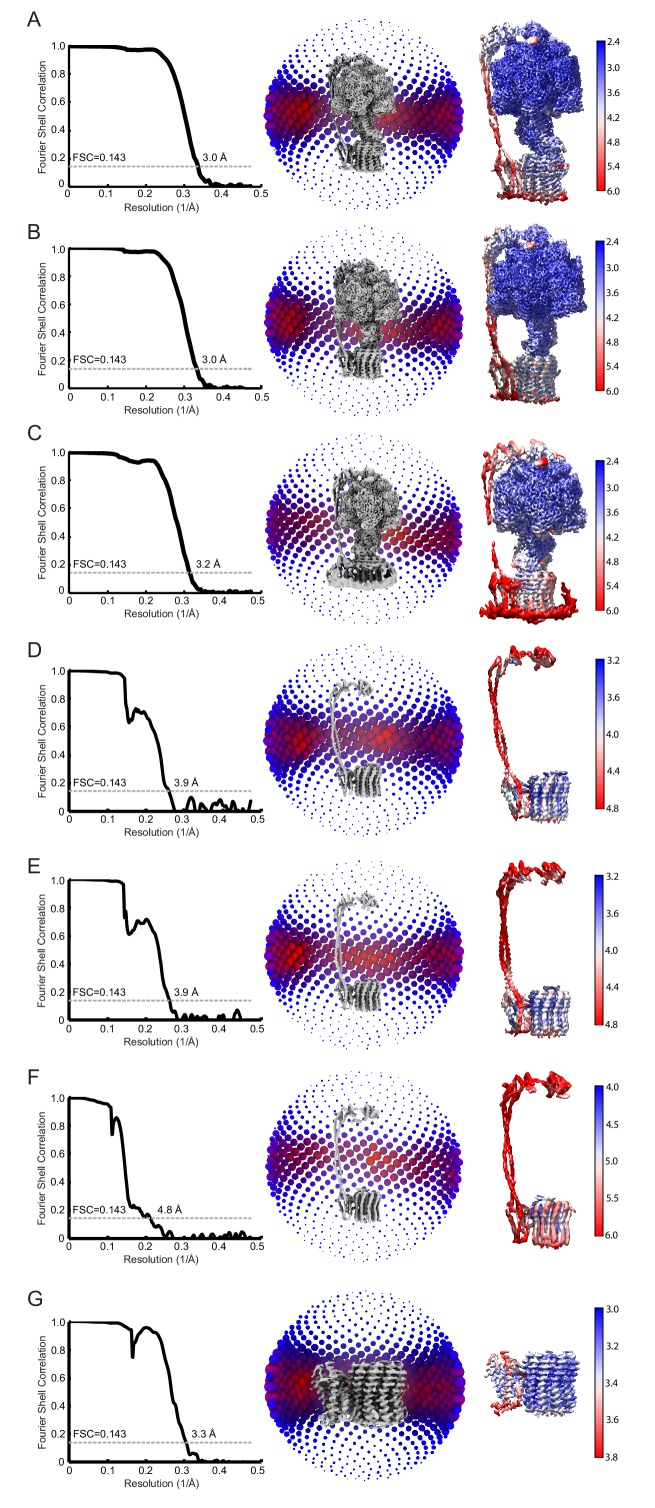
FSC, orientation distribution, and local resolution of the cryo-EM maps used to build atomic models. (**A** to **C**) Full maps of *Bacillus* PS3 ATP synthase class 1, 2, and 3. (**D** to **F**) Focused refinement maps of class 1, 2 and 3 including subunit *δ* and the F_O_ subunits. (**G**) Focused refinement map of the membrane-bound region only.

**Figure 1—figure supplement 4.**
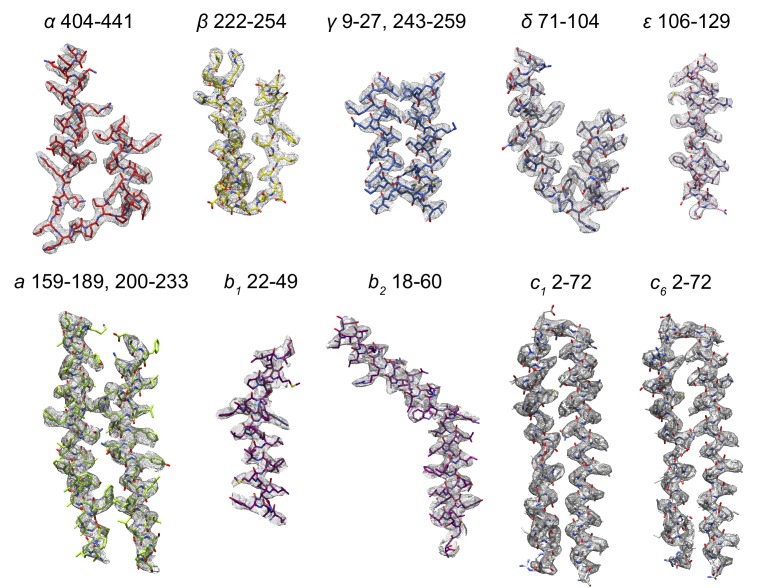
Examples of atomic models from subunits built in the experimental cryo-EM maps.

**Figure 1—figure supplement 5.**
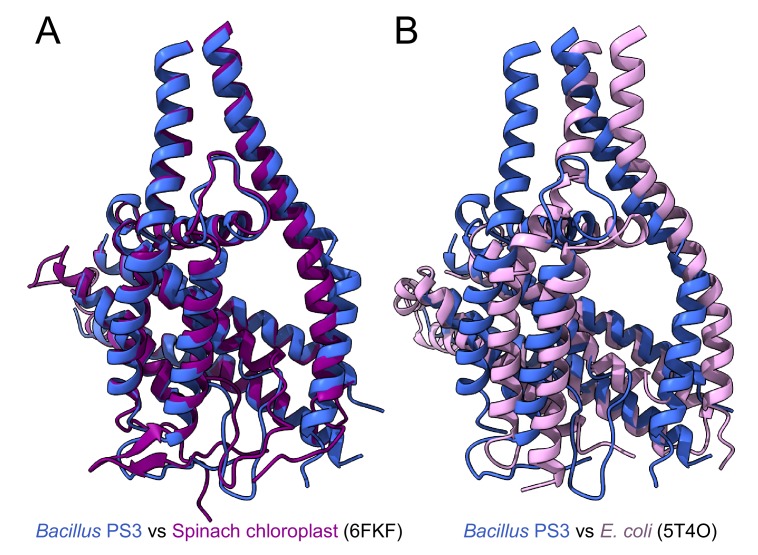
Comparison of *Bacillus* PS3 subunits ab_2_ with corresponding structures from spinach chloroplast and *E. coli*. Overlay of *Bacillus PS3* ATP synthase subunits *ab_2_* with spinach chloroplast ATP synthase subunit abb’ PDB 6FKF (Hahn et al., 2018) (**A**) and *E.coli* ATP synthase subunits ab_2_ PDB 5T4O (Sobti et al., 2016) (**B**).

## Results and discussion

### Structure determination and overall architecture

Subunits of *Bacillus* PS3 ATP synthase, including subunit *β* bearing an N-terminal 10 × His tag, were expressed from a plasmid in *E. coli* strain DK8, which lacks endogenous ATP synthase (Klionsky et al., 1984; Suzuki et al., 2002). The complex was extracted from membranes with detergent, purified by metal-affinity chromatography, and subjected to cryo-EM analysis (Figure 1—figure supplement 1). Three conformations corresponding to different rotational states of the enzyme were identified by ab-initio 3D classification and refined to high resolution. The 3D classes contain 45, 35, and 20% of particle images and the overall resolutions of the corresponding cryo-EM maps were 3.0, 3.0, and 3.2 Å, respectively (Figure 1—figure supplements 2 and 3). Estimation of local resolution suggests that the F_1_ regions of the maps, which are larger than the F_O_ regions and appear to dominate the image alignment process, are mostly at between 2.5 and 3.5 Å resolution, whereas the F_O_ regions were limited to lower resolution (Figure 1—figure supplement 3). Focused refinement (Bai et al., 2015) of the F_O_ region and peripheral stalk subunits *ab_2_c_10_* and *δ* (corresponding to the subunit *OSCP* in mitochondrial ATP synthase) improved the resolution of the F_O_ regions considerably for all three classes but not enough to resolve density for most of the amino acid side chains. An improved map of the F_O_ region was obtained by focused refinement of the membrane-embedded region only, excluding the soluble portion of subunit *b* with particle images from all three classes (Figure 1—figure supplement 2). Overall, amino acid side chain detail can be seen for subunits *α_3_, β_3_, γ, δ, ε, a, c_10_*-ring, and the transmembrane α-helices of *b_2_* (Figure 1—figure supplement 4). The soluble region of the two *b*-subunits was modeled as poly-alanine (Supplementary file 1).

The general architecture of the enzyme resembles *E. coli* ATP synthase (Sobti et al., 2016) and the more distantly related spinach chloroplast enzyme (Hahn et al., 2018) but with striking differences. As observed previously in a *Bacillus* PS3 F_1_-ATPase crystal structure (PDB 4XD7) (Shirakihara et al., 2015), the three catalytic *β* subunits adopt ‘open’, ‘closed’, and ‘open’ conformations, different from the ‘half-closed’, ‘closed’, and ‘open’ conformations seen in the auto-inhibited *E. coli* F_1_-ATPase (Cingolani and Duncan, 2011), and the ‘closed’, ‘closed’, and ‘open’ conformations seen in chloroplast ATP synthase (Hahn et al., 2018) and most mitochondrial ATP synthase structures (Abrahams et al., 1994; Stock et al., 1999). This difference, with the *half-closed β_DP_* of the *E. coli* enzyme appearing as *open* in the *Bacillus* PS3 enzyme, suggests species-specific differences in inhibition by subunit *ε* (Figure 1B, pink density), which inserts into the α/β interface and forces *β_DP_* into the *open* conformation.

Thermophilic proteins achieve stability at high temperature through mechanisms that include an increased number of ionic interactions, shorter loops between secondary structure elements, and tighter packing of hydrophobic regions (Jaenicke and Böhm, 1998; Kumar and Nussinov, 2001; Szilágyi and Závodszky, 2000). Comparison of individual subunit structures from the F_1_ regions of ATP synthases from thermophiles (*Bacillus PS3* and *Caldalaklibacillus thermarum* [PDB 5HKK] (Ferguson et al., 2016)) and mesophiles (*E. coli* [PDB 3OAA] (Cingolani and Duncan, 2011), *Paracoccus denitrificans* [5DN6] (Morales-Rios et al., 2015), and *Spinacia oleracea* chloroplast [PDB 6FKF] (Hahn et al., 2018)) did not show clear evidence of tighter packing or shorter loops in the complexes from thermophiles. However, there are more ionic interactions in the F_1_-ATPase structures from thermophiles than from mesophiles, suggesting that these interactions may play a role in stabilizing the complexes.

In the F_O_ region, one copy of subunit *b* is positioned at a location equivalent to that of the mitochondrial subunit *b*, while the second copy occupies the position of yeast subunit *8* (mammalian *A6L*) on the other side of subunit *a* (Figure 1B). Despite the different *c*-ring sizes (10 *c*-subunits in *Bacillus* PS3 versus 14 in spinach chloroplasts), the backbone positions of subunits *ab_2_* from *Bacillus* PS3 overlap with subunits *abb’* from spinach chloroplast ATP synthase (Hahn et al., 2018) (Figure 1—figure supplement 5A). Comparison of the atomic model of the F_O_ region from *Bacillus* PS3 and the backbone model of the *E. coli* complex from cryo-EM at ~7 Å resolution (PDB 5T4O) (Sobti et al., 2016) showed significant structural differences in transmembrane α-helices of subunit *b* relative to subunit *a* (Figure 1—figure supplement 5B). Rather than reflecting true differences between *E. coli* and *Bacillus* PS3 ATP synthase structures, these deviations are likely due to the lower resolution of the *E. coli* maps.

### Flexibility in the peripheral and central stalks

**Figure 2.**
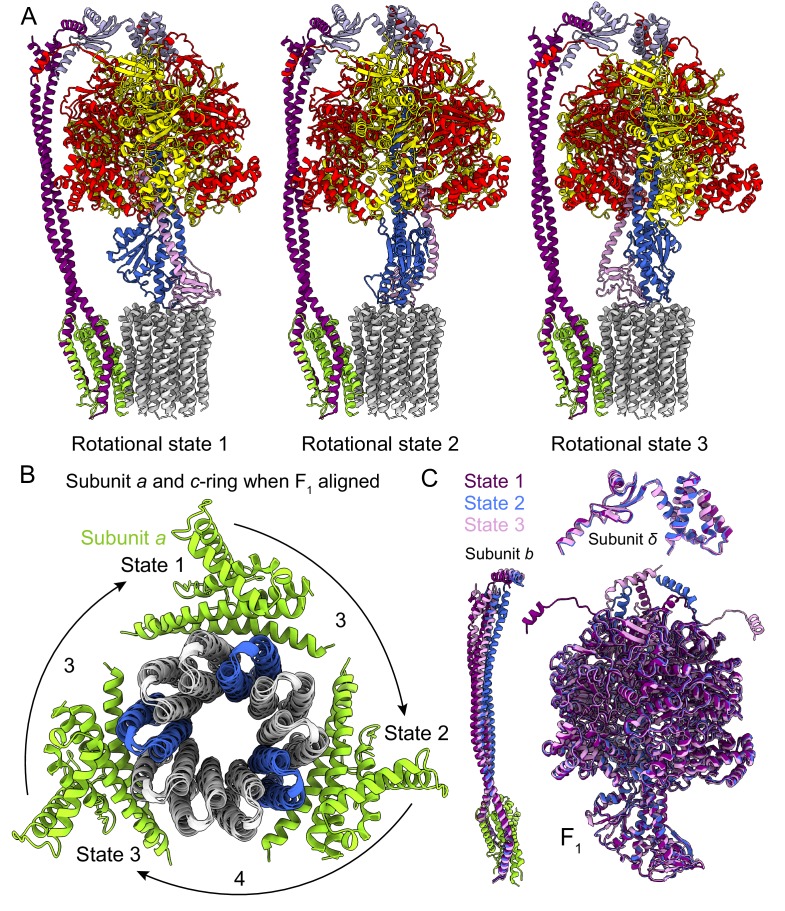
Rotational states of ATP synthase. (**A**) Atomic models of the three rotational states of *Bacillus* PS3 ATP synthase with subunits coloured the same as in Figure 1. (**B**) Top view of the *c*-ring and subunit *a* of the three rotational states from the cytoplasm when the F_1_ regions of the three states are aligned. Rotation steps of the complex between states are ~3, 4, and 3 *c*-subunits. (**C**) Comparison of the atomic models of subunits *b*, *δ*, and other F_1_ region subunits in the different rotational states. The *b* subunits appear to be the most flexible part of the enzyme.

As expected, the most striking difference between the three rotational states of the *Bacillus* PS3 structure is the angular position of the rotor (subunits *γεc_10_*) (Figure 2A, Video 1). The structure of the ATP synthase, with three *αβ* pairs in the F_1_ region and 10 *c*-subunits in the F_O_ region, results in symmetry mismatch between the 120° steps of the F_1_ motor and 36° steps of the F_O_ motor. The 120° steps of the F_1_ motor gives an average rotational step of 3.3 *c*-subunits, with the closest integer steps being 3, 4 and 3 *c*-subunits. By comparing the positions of equivalent *c*-subunits in different rotational states, the observed rotational step sizes in the three rotational states of the ATP synthase appear to be almost exactly 3, 4 and 3 *c*-subunits (Figure 2B). At the present resolution, the structures of subunit *a* and the *c-*ring do not appear to differ between rotary states. Similar integer step sizes were found in yeast ATP synthase (Vinothkumar et al., 2016) and V-ATPase (Zhao et al., 2015), which also contain 10 *c*-subunits. However, non-integer steps were seen in the chloroplast (14 *c*-subunits) (Hahn et al., 2018) and bovine (8 *c*-subunits) (Zhou et al., 2015) ATP synthases, indicating that the *c*-subunit steps between the rotational states of rotary ATPases likely depends on the number of *c*-subunits.

**Video 1.** Atomic models of the *Bacillus* PS3 ATP synthase in three rotational states.

The unequal number of *c*-subunit steps between rotational states or the different interactions made by the three *αβ* pairs with the *b_2_δ* peripheral stalk could lead to a variable rotation speed for the *c-*ring in the active enzyme, analogous to kinetic limping in kinesin motors (Asbury et al., 2003). Alternatively, flexibility in the enzyme could maintain a constant rotational velocity. Indeed, flexibility is thought to be important for the smooth transmission of power between the F_1_ and F_O_ regions, which often have mismatched symmetries (Wang and Oster, 1998; Pänke et al., 2001; Mitome et al., 2004). Earlier studies suggested that the central stalk (subunits *γ* and *ε* in bacteria) is the main region responsible for the transient storage of torsional energy in rotary ATPases (Sielaff et al., 2008; Wächter et al., 2011; Ernst et al., 2012; Okazaki and Hummer, 2015). Comparison of the three rotational states of the *Bacillus* PS3 enzyme also shows that C-terminal water-soluble part of subunit *b* displays the most significant conformational variability between states, while the subunits in the F_1_ region show little flexibility beyond the catalytic states of the *αβ* pairs (Figure 2C; Video 1). The structure of the yeast ATP synthase F_O_ dimer (Guo et al., 2017), which lacked the the F_1_ region and an intact peripheral stalk, showed that the *c*-ring and subunit *a* are held together by hydrophobic interactions rather than by the peripheral stalk. In *Bacillus* PS3 ATP synthase, the peripheral stalk is structurally simpler and more flexible than in yeast mitochondria (Srivastava et al., 2018), suggesting that the bacterial subunits *a* and the *c*-ring are also held together by hydrophobic interactions and not the peripheral stalk. Given that these structures represent resting states of the bacterial ATP synthase, additional subunits, such as those in the central stalk, may show flexibility while under strain during rotation.

### Nucleotide binding in the F_1_ region and inhibition by subunit ε

**Figure 3.**
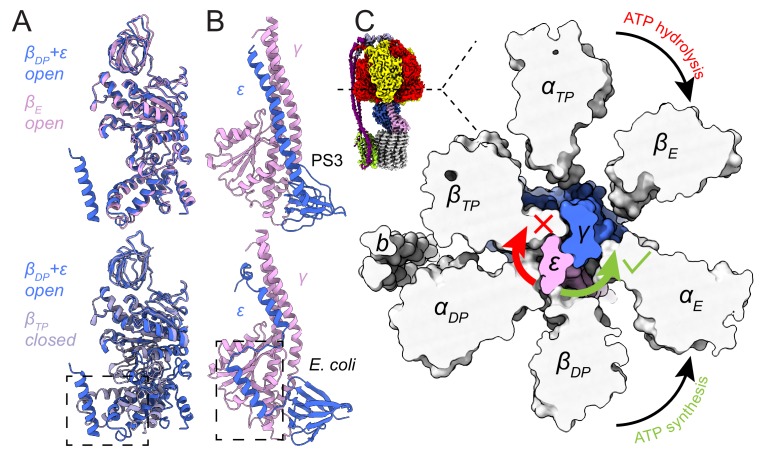
Inhibition of ATP hydrolysis by subunit *ε*. (**A**) Comparison of *β_DP_* (blue) with *β_E_* (pink, top) and *β_TP_* (light purple, bottom). *β_DP_* is forced to adopt an *open* conformation by subunit *ε* (dashed box). (**B**) Comparison of subunits *γ* (pink) and *ε* (blue) of ATP synthases from *Bacillus* PS3 (top) and *E. coli* (bottom, PDB 3OAA (Cingolani and Duncan, 2011)). The dashed box shows additional interaction between subunits *ε* and *γ* in the *E. coli* complex. (**C**) Cross-section through the catalytic F_1_ region of the *Bacillus* PS3 ATP synthase. Subunit *ε* (pink) in the rotor is blocked from rotating in the direction of ATP hydrolysis (clockwise) by *β_TP_* but is free to rotate in the direction of ATP synthesis (counterclockwise).

The structure of the F_1_ region of the intact *Bacillus* PS3 ATP synthase and the earlier crystal structure of the dissociated F_1_-ATPase (PDB 4XD7) (Shirakihara et al., 2015) both show that the three catalytic *β*-subunits (*β_E_*, *β_TP_*, and *β_D_*_P_) adopt ‘open’, ‘closed’, and ‘open’ conformations, respectively (Figure 3A). In the crystal structure, which was prepared in the presence of CyDTA (trans-1,2-Diaminocyclohexane-N, N, N′, N′-tetraacetic acid monohydrate) as a chelating agent, there was no nucleotide in the three noncatalytic sites of the three *α-*subunits and the only nucleotide in a catalytic site was an ADP molecule without a Mg^2+^ ion in the *β_TP_* site. In contrast, all three non-catalytic sites in the cryo-EM map are occupied by Mg-ATP, while a Mg-ADP molecule and a weak density tentatively assigned to phosphate are found in the *β_TP_* site and by the p-loop of *β_E_*, respectively. The presence of physiological Mg^2+^ ions and nucleotide occupancy (Nalin and Cross, 1982) in the cryo-EM map suggest that it shows a snapshot of the enzyme in the middle of its physiological catalytic cycle.

*Bacillus* PS3 ATP synthase is found in a conformation that has been proposed to allow ATP synthesis while ATP hydrolysis is auto-inhibited. In this state, subunit *ε* maintains an *up* conformation and inserts into the *α_DP_β_DP_* interface, forcing *β_DP_* to adopt an open conformation (Figure 3A, lower, dashed box) (Shirakihara et al., 2015). In the crystal structure (PDB 4XD7) (Shirakihara et al., 2015), the C-terminal sequence of subunit *ε* was modeled as two α-helical segments broken at Ser 106, while the cryo-EM structures show the C-terminal part is in fact entirely α-helical. In comparison, subunit *ε* from the auto-inhibited *E. coli* F_1_-ATPase structure (PDB 3OAA) (Cingolani and Duncan, 2011) maintains its two C-terminal α-helices (Figure 3B), with its *β_DP_* adopting a *half-closed* conformation that binds to Mg-ADP. The C-terminal α-helix of the *E. coli* subunit *ε* inserts slightly deeper into the *α_DP_β_DP_* interface but overall in a manner similar to that of the *Bacillus* PS3 subunit *ε*. However, the second α-helix in *E. coli* is offset by a 10-residue loop that allows it to interact with subunit *γ*. This interaction (Figure 3B, lower, dashed box) may stabilize the *up* conformation of subunit *ε* in *E. coli*, explaining why auto-inhibition in *E. coli* does not depend on ATP concentration (Laget and Smith, 1979; Sekiya et al., 2010) while in *Bacillus* PS3 it does. Interestingly, during ATP synthesis, *Bacillus* PS3 subunit *ε* was proposed to maintain the *up* conformation (Suzuki et al., 2003), suggesting that it only blocks ATP hydrolysis but not ATP synthesis. For a canonical ATP synthase, the substrates ADP and Pi bind to an *open β_E_*. The *β_E_* subsequently transitions to become the *closed β_DP_* and then *β_TP_*, driven by rotation of the central rotor, producing an ATP molecule that is ultimately released when the *closed β_TP_* converts back to an open *β_E_* (Abrahams et al., 1994). For the *Bacillus* PS3 ATP synthase to produce ATP with subunit *ε* in the *up* conformation, substrate would need to bind to the *β_DP_* site instead of the usual *β_E_* site, with an ATP molecule produced on transition to a *closed β_T_*_P_. The cryo-EM maps show that a clash between subunit *ε* and *β_T_*_P_ blocks the central rotor turning in the direction of ATP hydrolysis while it is still free to turn in the direction of ATP synthesis (Figure 3C), which could explain the ability of subunit *ε* to selectively inhibit ATP hydrolysis (Suzuki et al., 2003).

### Subunit organization in the F_O_ region

**Figure 4.**
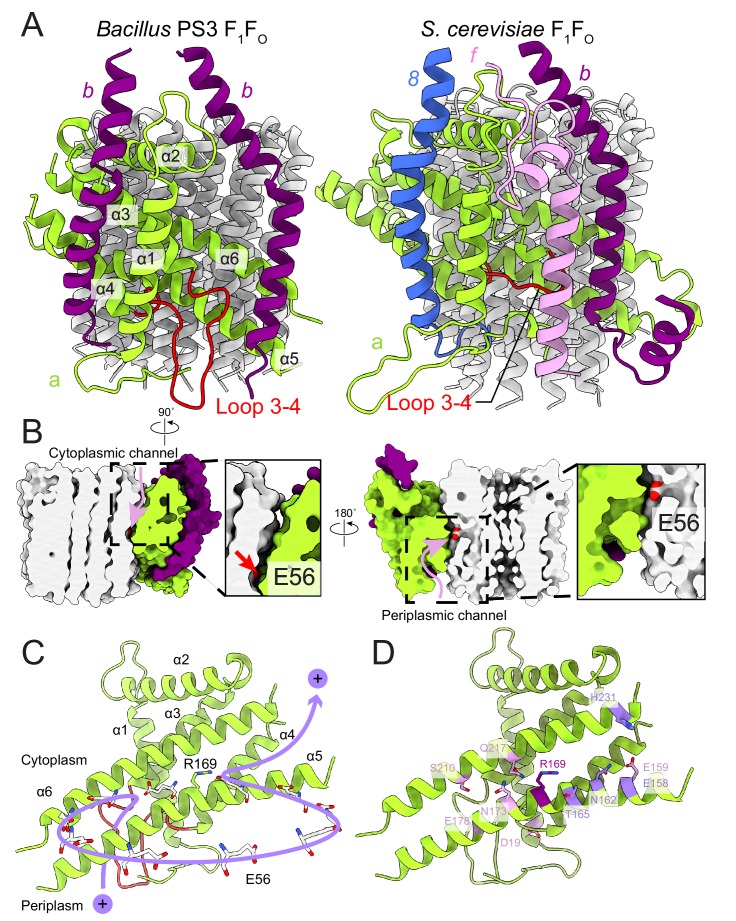
F_O_ region subunits and proton translocation in *Bacillus* PS3 ATP synthase. (**A**) Comparison of the F_O_ regions from *Bacillus* PS3 (left) and *S. cerevisiae* (right). (**B**) Cross sections through a surface representation of the F_O_ region (simulated with rolling of a 1.4 Å sphere (Goddard et al., 2018)) show the cytoplasmic (left) and periplasmic (right) proton half-channels. (**C**) Proton translocation pathway of *Bacillus* PS3 ATP synthase. During ATP synthesis, a proton enters the complex via the periplasmic half-channel, passing between α-helices 5 and 6 of subunit *a* to bind to the Glu 56 residue of a subunit *c*. The proton then rotates with the *c*-ring until it reaches the cytoplasmic half-channel formed between subunit *a* and the *c*-ring. In the cytoplasmic half-channel, the proton is released from the Glu residue due to its interaction with the positively charged Arg 169 of subunit *a*. A Glu 56 residue from each protomer of the *c*-ring is shown. (**D**) Subunit *a* of *Bacillus* PS3 ATP synthase. Arg 169 is in purple, important residues for proton translocation identified by mutagenesis in *E. coli* ATP synthase are in pink, and other residues that appears to contribute to proton transfer in the cytosolic proton half-channel are in light purple.

In the bacterial ATP synthase structure, the F_O_ subunits *ab_2_* display an organization similar to the yeast F_O_ complex (PDB 6B2Z, Figure 4A) (Guo et al., 2017). Subunit *a* and the first copy of subunit *b* occupy the same positions as their yeast counterparts, while the second copy of subunit *b* is found at a position equivalent to subunit 8 in the yeast enzyme, which is known as *A6L* in mammals. Atomic models for ATP synthase from mitochondria (Guo et al., 2017; Klusch et al., 2017; Srivastava et al., 2018) and chloroplasts (Hahn et al., 2018) support the idea that transmembrane proton translocation in ATP synthases occurs via two offset half-channels formed by subunit *a* (Vik and Antonio, 1994; Junge et al., 1997). Subunit *a* from *Bacillus* PS3 shares 21.0% and 29.1% sequence identity with its yeast and chloroplast homologs, respectively, and the atomic model shows that the folding of these homologs is mostly conserved (Figure 4—figure supplement 1). Multi-sequence alignment of subunit *a* from different species indicates that bacterial and chloroplast subunit *a* contain a larger periplasmic loop between α-helices 3 and 4 than found in the mitochondrial subunit (Figure 4A, left; Figure 4—figure supplement 2). The sequence for this loop varies significantly among species, suggesting that it is unlikely to be involved in the core function of proton translocation, despite being proximal to the periplasmic proton half-channel. Yeast and mammalian mitochondrial ATP synthases contain subunit *f*, which has a transmembrane α-helix adjacent to the transmembrane α-helix 1 of subunit *a* (Figure 4A, right), anchoring subunit *b* between α-helices 5 and 6 of subunit *a*. The location of the loop between α-helices 3 and 4 of the *Bacillus* PS3 subunit *a* suggests that it serves a similar structural role, compensating for the lack of subunit *f* in bacteria. The loop forms an additional interface with subunit *b* near the periplasmic side of the membrane region and may interact with the N terminus of subunit *b* in the periplasm as well. Two interfaces are also present between the second copy of subunit *b* and subunit *a*, one with the first transmembrane α-helix, and the other with the hairpin of α-helices 3 and 4 (Figure 4A). The structure suggests that two interfaces are necessary for subunits *a* and *b* to maintain a stable interaction.

**Figure 4—figure supplement 1.**
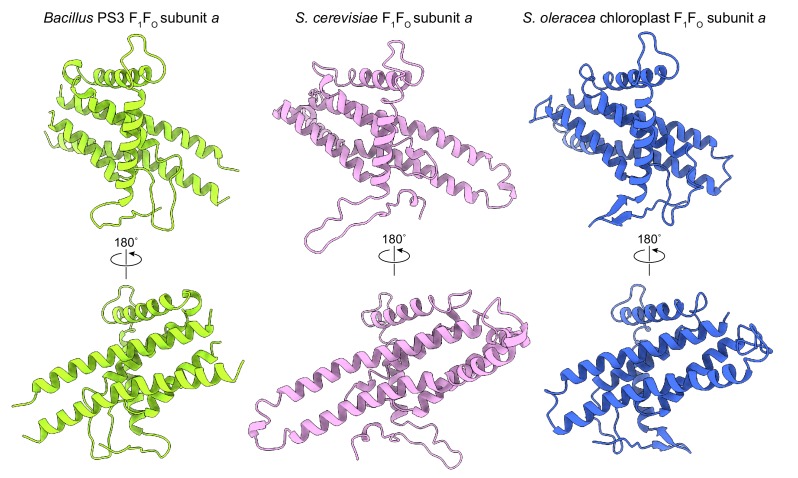
Comparison of subunit *a* structures from different organisms. (**A**) *Bacillus* PS3 ATP synthase. (**B**) *S. cerevisiae* mitochondrial ATP synthase (PDB 6B2Z (Guo et al., 2017)). (**C**) *S. oleracea* chloroplast ATP synthase (PDB 6FKF (Hahn et al., 2018)).

**Figure 4—figure supplement 2.**
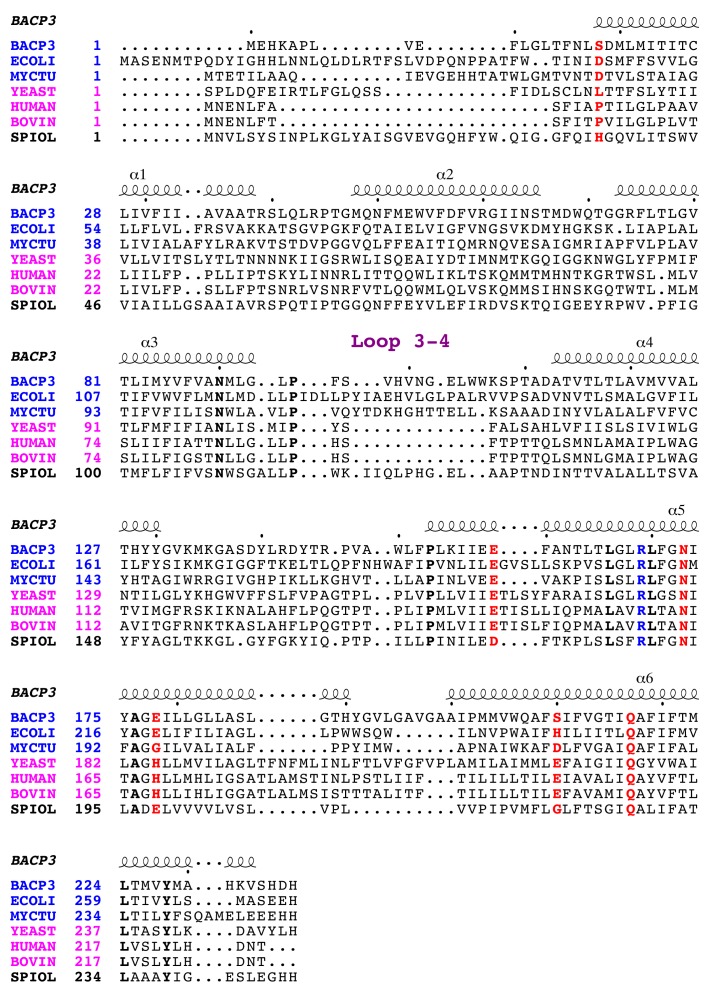
Multiple sequence alignment of subunit *a*. Sequences are from bacteria (blue), mitochondria (pink), and chloroplast (black), including *Bacillus* PS3 (BACP3), *E. coli* (ECOLI), *Mycobacterium tuberculosis* (MYCTU), *S. cerevisiae* (YEAST), *Homo sapiens* (HUMAN), *Bos taurus* (BOVINE), and *Spinacia oleracea* (SPIOL). The functionally important Arg residues for proton translocation are in blue. Other important residues for proton translocation identified by mutagenesis in *E. coli* ATP synthase are in red. Strictly conserved residues are in bold. Figure was generated with ESPript 3.0 (Robert and Gouet, 2014).

**Figure 4—figure supplement 3.**
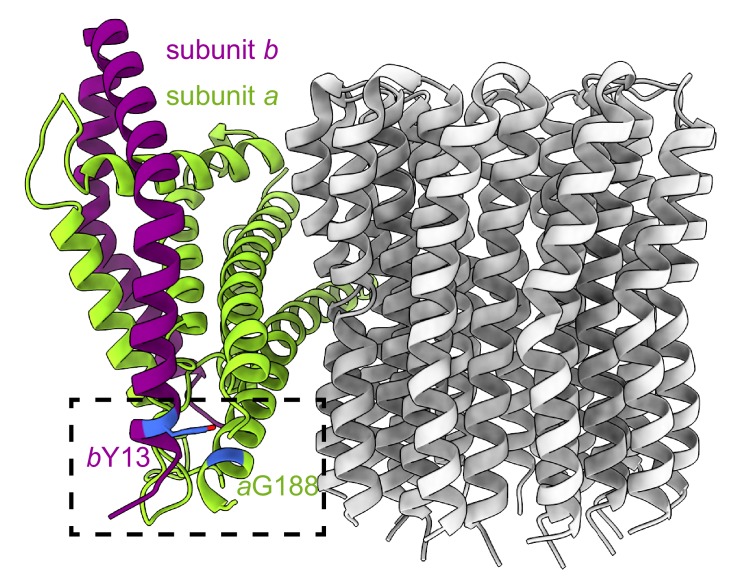
Positions of subunit *b* Tyr 13 and subunit *a* Gly 188. Side view of the F_O_ subunits. The residues of interest are inside the dashed box and are coloured in blue.

### Proton translocation through the F_O_ region

The *Bacillus* PS3 ATP synthase structure implies a path for proton translocation through the bacterial complex involving two half-channels similar to the paths described for the mitochondrial and chloroplast enzymes. The cytoplasmic half-channel consists of an aqueous cavity at the interface of subunit *a* and the *c*-ring (Figure 4B, left). The periplasmic half-channel is formed from a cavity between α-helices 1, 3, 4 and 5 of subunit *a*, and reaches the *c*-ring via a gap between α-helices 5 and 6 (Figure 4B, right). In the atomic model, both channels are visible when modeling the surface with a 1.4 Å sphere that mimics a water molecule (Goddard et al., 2018) (Figure 4B). The channels are wide and hydrophilic, suggesting that water molecules could pass freely through each of the channels before accessing the conserved Glu 56 of the *c*-subunits. During ATP synthesis, protons travel to the middle of the *c*-ring via the periplasmic half-channel and bind to the Glu 56 residue of a subunit *c* (Figure 4C). Protonation of the glutamate allows rotation of the ring counter-clockwise, when viewed from F_1_ toward F_O_, delivering the subunit *c* into the hydrophobic lipid bilayer. Protonation of the remaining nine subunits in the *c*-ring returns the first glutamate to subunit *a*, now into the cytoplasmic half-channel, where it releases its proton to the cytoplasm due to interaction with the positively charged Arg 169 of subunit *a*. The proposed channels are consistent with a series of experiments probing water accessibility of residues in the *E. coli* ATP synthase subunit *a* by mutating them to cysteines and testing their accessibility by Ag^+^ ions (Angevine et al., 2003; Angevine and Fillingame, 2003; Angevine et al., 2007; Moore et al., 2008). Residues that are close to the *c*-ring, such as S202, S206, N214, and I249 (A161, T165, N173, and G214 in *Bacillus PS3*) are among the most accessible by Ag^+ 52^, suggesting that the half-channels do not exclude Ag^+^. Therefore, it is likely that Na^+^ ions, which are similar in size to Ag^+^ ions, are also not excluded by subunit *a*. It is also known that the *c*-rings from Na^+^- and H^+^-driven ATP synthases have different affinities for Na^+^ and H (Krah et al., 2010; Leone et al., 2015; Mayer et al., 2012; Schlegel et al., 2012), and that the *c*-ring of the Na^+^-driven ATP synthase from *llyobacter tartaricus* does not bind K^+^ or Cs^+^ ions (Leone et al., 2015). Together, these results suggest that ion selectivity in ATP synthases is probably determined by the *c*-ring, not subunit *a*.

In eukaryotes, subunit *a* is encoded by the mitochondrial genome, limiting genetic interrogation of the roles of different residues. In contrast, numerous mutagenesis studies have been performed on bacterial subunits *a* and *b*, with *E. coli* ATP synthase being the most frequently studied (Vik et al., 2000; Cain, 2000). A single G9D mutation in the *E. coli* subunit *b* (positionally equivalent to Y13D in *Bacillus* PS3), results in assembled but non-functional ATP synthase (Porter et al., 1985), while multiple N-terminal mutations in subunit *b* can either disrupt enzyme assembly or ATP hydrolysis (Hardy et al., 2003). In *Bacillus* PS3, Tyr 13 is part of the transmembrane α-helix of subunit *b* and is adjacent to Gly 188 of subunit *a* (Figure 4—figure supplement 3, dashed box). In *E. coli* subunit a, Gly 188 is replaced by a leucine (Leu 229). Therefore, the G9D mutation in *E. coli* not only introduces a charged residue into a hydrophobic transmembrane α-helix, but also creates a steric clash with Leu 229 of subunit *a*, explaining why the mutation leads to an inactive enzyme. Remarkably, the single N-terminal membrane-embedded α-helix in each of the two copies of subunit *b* in the *Bacillus* PS3 ATP synthase forms different interactions with subunit *a* (Figure 4A). One surface interacts with transmembrane α-helices 1, 2, 3, and 4 of subunit *a* while the other interacts with α-helices 5 and 6 and the loop between α-helices 3 and 4 of subunit *a*. Given that the N-terminal α-helix of subunit *b* makes interactions with different regions of subunit *a*, it is not surprising that mutations in this region are often detrimental to the assembly and activity of the complex. Cross-linking experiments suggested that the N terminus of the two copies of subunit *b* are in close proximity to each other (Dmitriev et al., 1999). However, the atomic model shows that the transmembrane α-helix of the *b*-subunits are on opposite sides of subunit *a*, suggesting that the cross-linking results may be due to non-specific interactions of *b*-subunits from neighboring ATP synthases.

In *E. coli*, Arg 210 of subunit *a* (Arg 169 in *Bacillus* PS3) tolerates the fewest mutations (Cain and Simoni, 1989; Hatch et al., 1995; Lightowlers et al., 1987; Ishmukhametov et al., 2008). Recent structures of rotary ATPases suggest that the importance of this residue derives from its role in releasing protons bound to the Glu residues of the *c*-subunits as they enter the cytoplasmic half-channel, as well as preventing short-circuiting of the proton path by protons flowing between half-channels without rotation of the *c*-ring (Morales-Rios et al., 2015; Zhao et al., 2015; Zhou et al., 2015; Allegretti et al., 2015; Mazhab-Jafari et al., 2016). Other residues in the *E. coli* subunit *a* identified by mutation as being functionally important include Glu 196 (Glu 159 in *Bacillus* PS3) (Vik et al., 1988; Lightowlers et al., 1988), Glu 219 (Glu 178) (Vik et al., 1988; Lightowlers et al., 1988; Eya et al., 1991), His 245 (Ser 210) (Lightowlers et al., 1987; Cain and Simoni, 1986; Cain and Simoni, 1988), Asp 44 (Asp 19) (Howitt et al., 1990), Asn 214 (Asn 173) (Cain and Simoni, 1989), and Gln 252 (Gln 217) (Eya et al., 1991; Hartzog and Cain, 1993) (Figure 4D). When mapped to the *Bacillus* PS3 structure, only Glu 196 (Glu 159 in *Bacillus* PS3) is close to the cytoplasmic half-channel. Extensive mutations of *E. coli* Glu 196 showed that enzyme activity depends on the charge and polarity of the residue with Glu > Asp > Gln = Ser = His > Asn > Ala > Lys (Vik et al., 1988). Therefore, the negative surface charge from Glu 196 (Glu 159) near the cytoplasmic half-channel facilitates proton transport across the lipid bilayer. The atomic model of subunit *a* also suggests that other residues such as *Bacillus* PS3 Thr 165, Asn 162, Glu 158, Tyr 228, and His 231, which are close to the cytoplasmic half-channel, may contribute to channel formation. Many functional residues identified by mutagenesis are clustered around the periplasmic half-channel. In the atomic model of the *Bacillus* PS3 subunit *a*, Asp 19 and Glu 178 are close to the periplasm, while Ser 210, Asn 173, and Gln 217 are deeper inside the membrane. Among these residues, Glu 178 and Ser 210 are considered to be more important to enzyme function than Asn 173 and Gln 217, as mutations of corresponding residues in *E. coli* are more likely to abolish the proton translocation by the complex (Vik et al., 2000). The Glu 219/His 245 residue pair in *E. coli* (Cain and Simoni, 1988) also occur in the *S. cerevisiae* (His 185/Glu 223) and human (His 168/Glu 203) mitochondrial ATP synthases (Figure 4—figure supplement 2). These residues do not appear to be close enough to form a hydrogen bond in the *S. cerevisae* F_O_ dimer structure (Guo et al., 2017). In *Bacillus PS3* subunit *a*, the His residue is replaced by a serine (Ser 210) that similarly does not appear to close enough to Glu 178 to form a hydrogen bond. Interestingly, although many of these functional residues appear important, their mutation to amino acids that cannot be protonated or deprotonated often does not completely abolish proton translocation (Vik et al., 2000). The atomic model of *Bacillus* PS3 subunit *a* shows that the proton half-channels are wide enough for water molecules to pass through freely. This observation suggests that the function of these conserved polar and charged residues is not the direct transfer of protons during translocation. Rather, their presence may help maintain a hydrophilic environment for water-filled proton channels. This role allows different species to use unique sets of polar and charged residues to form their proton half-channels. This variability suggests a remarkably flexible proton translocation mechanism for this highly efficient macromolecular machine.

## Materials and methods

### Protein expression and purification

*E. coli* strain DK8, in which the genes encoding endogenous ATP synthase subunits were deleted (Klionsky et al., 1984), was transformed with plasmid pTR-ASDS (Suzuki et al., 2002) encoding *Bacillus* PS3 ATP synthase with a 10 × His tag at the N terminus of subunit *β*. Transformed *E. coli* cells were grown in 2 × TY medium at 37°C for 20 hr before being harvested by centrifugation at 5400 g. Cell pellets were resuspended in lysis buffer (50 mM Tris-HCl pH 7.4, 5 mM MgCl_2_, 10% [w/v] glycerol, 5 mM 6-aminocaproic acid, 5 mM benzamidine, 1 mM PMSF) and lysed with three passes through an EmulsiFlex-C3 homogenizer (Avestin) at 15 to 20 kbar. All protein preparation steps were performed at 4°C unless otherwise stated. Cell debris was removed at 12,250 g for 20 min, and the cell membrane fraction was collected by centrifugation at 184,000 g for 1 hr. Membranes were washed twice with lysis buffer before being resuspended in solubilization buffer (50 mM Tris-HCl pH 7.4, 10% [w/v] glycerol, 250 mM sucrose, 5 mM 6-aminocaproic acid, 5 mM benzamidine, 1 mM PMSF) and solubilized by the addition of glycol-diosgenin (GDN) to 1% (w/v) and mixing for 1 hr at room temperature. Insoluble material was removed by centrifugation at 184,000 g for 45 min and solubilized membranes were loaded onto a 5 ml HisTrap HP column (GE Healthcare) equilibrated with buffer A (solubilization buffer with 20 mM imidazole, 300 mM sodium chloride, and 0.02% [w/v] GDN). The column was washed with five column volumes of buffer A, and ATP synthase was eluted with three column volumes of buffer B (buffer A with 200 mM imidazole). Fractions containing ATP synthase were pooled and concentrated prior to being loaded onto a Superose 6 increase 10/300 column (GE Healthcare) equilibrated with gel filtration buffer (20 mM Tris-HCl pH 7.4, 5 mM MgCl_2_, 10% [w/v] glycerol, 150 mM sodium chloride, 5 mM 6-aminocaproic acid, 5 mM benzamidine, 0.02% [w/v] GDN). The peak corresponding to *Bacillus* PS3 ATP synthase was pooled and concentrated to ~10 mg/ml prior to storage at −80°C.

### Cryo-EM and image analysis

Prior to grid freezing, glycerol was removed from samples with a Zeba spin desalting column (Thermo Fisher Scientific). Purified ATP synthase (2.5 μL) was applied to homemade nanofabricated EM grids (Marr et al., 2014) consisting of a holey layer of gold (Russo and Passmore, 2014; Meyerson et al., 2014) that had been glow-discharged in air for 2 min. Grids were then blotted on both sides in a FEI Vitrobot mark III for 26 s at 4°C and ~100% RH before freezing in a liquid ethane/propane mixture (Tivol et al., 2008). Cryo-EM data were collected with a Titan Krios G3 electron microscope (Thermo Fisher Scientific) operated at 300 kV equipped with a Falcon 3EC direct detector device camera automated with *EPU* software. Data were recorded as 60 s movies at 2 s per frame with an exposure rate of 0.8 electron/pixel/s, and a calibrated pixel size of 1.06 Å.

All image processing steps were performed in *cryoSPARC v2* (Punjani et al., 2017) unless otherwise stated. 10,940 movies were collected. Movie frames were aligned with an implementation of *alignframes_lmbfgs* within *cryoSPARC v2* (Rubinstein and Brubaker, 2015) and CTF parameters were estimated from the average of aligned frames with *CTFFIND4* (Rohou and Grigorieff, 2015). 1,866,804 single particle images were selected from the aligned frames with *Relion 2.1* (Fernandez-Leiro and Scheres, 2017) and beam-induced motion of individual particles corrected with an improved implementation of *alignparts_lmbfgs* within *cryoSPARC v2* (Rubinstein and Brubaker, 2015). A subset of 1,238,140 particle images were selected by 2D classification in *cryoSPARC v2*. After initial rounds of ab-initio 3D classification and heterogeneous refinement, three classes corresponding to three main rotational states of the enzyme were identified, containing 405,432, 314,448, and 175,694 particles images (Figure 1—figure supplement 2). These 3D classes were refined with non-uniform refinement to overall resolutions of 3.0 Å, 3.0 Å and 3.2 Å, respectively, with the F_1_ region reaching higher resolution than the F_O_ region of the complex as seen from estimation of local resolution (Figure 1—figure supplement 3). Masked refinement with signal subtraction (focused refinement) (Bai et al., 2015) around subunits *ab_2_c_10_δ* excluding the detergent micelle improved the map quality of the membrane-embedded region as well as the peripheral stalk for all three classes. The membrane-embedded region (subunits *ac_10_* and transmembrane α-helices of the *b*-subunits) was improved further by focused refinement with particle images from all three classes, yielding a map at 3.3 Å resolution. All Fourier shell correlation (FSC) curves were calculated with independently refined half-maps and resolution was assessed at the 0.143 criterion with correction for the effects of masking maps. For illustration purposes, composite maps for each of the three rotational states were generated by combining the F_1_ region of the maps from non-uniform refinement, the peripheral stalk region from the maps obtained with focused refinement of subunits *ab_2_c_10_δ*, and the map from focused refinement of the membrane-embedded region. Specifically, each map was multiplied by a mask surrounding the region of interest and the resulting maps were adjusted to similar absolute grey scale by multiplying with a constant with *relion_image_handler* before being merged with the maximum function volume operation in *UCSF Chimera* (Pettersen et al., 2004). These composite maps were not used for model refinement.

### Model building and refinement

Atomic models for subunits *α_3_β_3_γεδ* from all three rotational states were built with *Coot* (Emsley and Cowtan, 2004) into the maps of the intact complex from non-uniform refinement using PDB 4XD7 (Shirakihara et al., 2015) and PDB 6FKF (Hahn et al., 2018) as initial models for subunits *α_3_β_3_γε* and subunit *δ*, respectively. Subunits *ac_10_* and the membrane-embedded regions of subunits *b_2_* were built de novo in the 3.3 Å map of the membrane-embedded region of the complex from focused refinement. Backbone models of the soluble region of subunits *b_2_* for all three conformations were built with the maps from focused refinement of the peripheral stalk. Models were refined into their respective maps with *phenix.real_space_refine* (Adams et al., 2010) using secondary structure and geometric restraints followed by manual adjustments in *Coot* (Supplementary file 1). The quality of the models was evaluated by *MolProbity* (Chen et al., 2010) and *EMRinger* (Barad et al., 2015). To generate full models for all three rotational states, the model of subunits *ac_10_* and the membrane region of subunit *b_2_* were fit into the full maps of each conformation as three rigid bodies (*a*, *c_10_*, and *b_2_* membrane region) with *phenix.real_space_refine*. For classes 1 and 3, the backbone models of the soluble region of subunit *b_2_* did not fit the full maps well, and thus the fit was improved by molecular dynamics flexible fitting (MDFF) (Trabuco et al., 2008). The final composite model for each rotational state was generated by combining the models of subunits *α_3_β_3_γεδ*, the rigid body refined subunits *ac_10_* and membrane region of *b_2_*, and the backbone model of the soluble region of *b_2_*. Figures and movie were generated with Chimera (Pettersen et al., 2004) and ChimeraX (Goddard et al., 2018).

## Additional files

Supplementary file 1. Cryo-EM data acquisition, processing, atomic model statistics, and map/model depositions. (**A**) Cryo-EM data acquisition and image processing. (**B**) Map and model statistics. (**C**) Residues included in atomic models. (**D**) Deposited maps and associated coordinate files.

Transparent reporting form

### Data availability

CryoEM maps have been deposited in EMDB and atomic models in PDB.

The following datasets were generated:

GuoHRubinsteinJL2018Intact class 1Electron Microscopy Data BankEMD-9333

GuoHRubinsteinJL2018Intact class 1Protein Data Bank6N2Y

GuoHRubinsteinJL2018Intact class 2Electron Microscopy Data BankEMD-9334

GuoHRubinsteinJL2018Intact class 2Protein Data Bank6N2Z

GuoHRubinsteinJL2018Intact class 3Electron Microscopy Data BankEMD-9335

GuoHRubinsteinJL2018Intact class 3Protein Data Bank6N30

GuoHRubinsteinJL2018Focused Fo/stalk class 1Electron Microscopy Data BankEMD-9336

GuoHRubinsteinJL2018Focused Fo/stalk class 2Electron Microscopy Data BankEMD-9337

GuoHRubinsteinJL2018Focused Fo/stalk class 3Electron Microscopy Data BankEMD-9338

GuoH2018Focused FoProtein Data Bank6N2D

GuoHRubinsteinJL2018Focused FoElectron Microscopy Data BankEMD-9327

## References

[bib1] Abrahams JP, Leslie AG, Lutter R, Walker JE (1994). Structure at 2.8 A resolution of F1-ATPase from bovine heart mitochondria. Nature.

[bib2] Adams PD, Afonine PV, Bunkóczi G, Chen VB, Davis IW, Echols N, Headd JJ, Hung LW, Kapral GJ, Grosse-Kunstleve RW, McCoy AJ, Moriarty NW, Oeffner R, Read RJ, Richardson DC, Richardson JS, Terwilliger TC, Zwart PH (2010). PHENIX: a comprehensive Python-based system for macromolecular structure solution. Acta Crystallographica Section D Biological Crystallography.

[bib3] Allegretti M, Klusch N, Mills DJ, Vonck J, Kühlbrandt W, Davies KM (2015). Horizontal membrane-intrinsic α-helices in the stator a-subunit of an F-type ATP synthase. Nature.

[bib4] Angevine CM, Herold KA, Fillingame RH, Kaback HR (2003). Aqueous access pathways in subunit a of rotary ATP synthase extend to both sides of the membrane. PNAS.

[bib5] Angevine CM, Herold KA, Vincent OD, Fillingame RH (2007). Aqueous access pathways in ATP synthase subunit a. reactivity of cysteine substituted into transmembrane helices 1, 3, and 5. The Journal of Biological Chemistry.

[bib6] Angevine CM, Fillingame RH (2003). Aqueous access channels in subunit a of rotary ATP synthase. The Journal of Biological Chemistry.

[bib7] Asbury CL, Fehr AN, Block SM (2003). Kinesin moves by an asymmetric hand-over-hand mechanism. Science.

[bib8] Bai XC, Rajendra E, Yang G, Shi Y, Scheres SH (2015). Sampling the conformational space of the catalytic subunit of human γ-secretase. eLife.

[bib9] Barad BA, Echols N, Wang RY, Cheng Y, DiMaio F, Adams PD, Fraser JS (2015). EMRinger: side chain-directed model and map validation for 3D cryo-electron microscopy. Nature Methods.

[bib10] Cain BD (2000). Mutagenic analysis of the F0 stator subunits. Journal of Bioenergetics and Biomembranes.

[bib11] Cain BD, Simoni RD (1986). Impaired proton conductivity resulting from mutations in the a subunit of F1F0 ATPase in Escherichia coli. The Journal of Biological Chemistry.

[bib12] Cain BD, Simoni RD (1988). Interaction between Glu-219 and His-245 within the a subunit of F1F0-ATPase in Escherichia coli. The Journal of Biological Chemistry.

[bib13] Cain BD, Simoni RD (1989). Proton translocation by the F1F0ATPase of Escherichia coli. mutagenic analysis of the a subunit. The Journal of Biological Chemistry.

[bib14] Chen VB, Arendall WB, Headd JJ, Keedy DA, Immormino RM, Kapral GJ, Murray LW, Richardson JS, Richardson DC (2010). MolProbity: all-atom structure validation for macromolecular crystallography. Acta Crystallographica Section D Biological Crystallography.

[bib15] Cingolani G, Duncan TM (2011). Structure of the ATP synthase catalytic complex (F(1)) from Escherichia coli in an autoinhibited conformation. Nature Structural & Molecular Biology.

[bib16] Davies KM, Anselmi C, Wittig I, Faraldo-Gómez JD, Kühlbrandt W (2012). Structure of the yeast F1Fo-ATP synthase dimer and its role in shaping the mitochondrial cristae. PNAS.

[bib17] Dickson VK, Silvester JA, Fearnley IM, Leslie AG, Walker JE (2006). On the structure of the stator of the mitochondrial ATP synthase. The EMBO Journal.

[bib18] Dmitriev O, Jones PC, Jiang W, Fillingame RH (1999). Structure of the membrane domain of subunit b of the Escherichia coli F0F1 ATP synthase. The Journal of Biological Chemistry.

[bib19] Emsley P, Cowtan K (2004). Coot: model-building tools for molecular graphics. Acta crystallographica. Section D, Biological crystallography.

[bib20] Ernst S, Düser MG, Zarrabi N, Dunn SD, Börsch M (2012). Elastic deformations of the rotary double motor of single FoF1-ATP synthases detected in real time by förster resonance energy transfer. Biochimica Et Biophysica Acta (BBA) - Bioenergetics.

[bib21] Eya S, Maeda M, Futai M (1991). Role of the carboxyl terminal region of H(+)-ATPase (F0F1) a subunit from Escherichia coli. Archives of Biochemistry and Biophysics.

[bib22] Ferguson SA, Cook GM, Montgomery MG, Leslie AG, Walker JE (2016). Regulation of the thermoalkaliphilic F1-ATPase from *Caldalkalibacillus thermarum*. PNAS.

[bib23] Fernandez-Leiro R, Scheres SHW (2017). A pipeline approach to single-particle processing in RELION. Acta Crystallographica Section D Structural Biology.

[bib24] Goddard TD, Huang CC, Meng EC, Pettersen EF, Couch GS, Morris JH, Ferrin TE (2018). UCSF ChimeraX: meeting modern challenges in visualization and analysis. Protein Science.

[bib25] Guo H, Bueler SA, Rubinstein JL (2017). Atomic model for the dimeric F_O_ region of mitochondrial ATP synthase. Science.

[bib26] Hahn A, Vonck J, Mills DJ, Meier T, Kühlbrandt W (2018). Structure, mechanism, and regulation of the chloroplast ATP synthase. Science.

[bib27] Hardy AW, Grabar TB, Bhatt D, Cain BD (2003). Mutagenesis studies of the F1F0 ATP synthase b subunit membrane domain. Journal of Bioenergetics and Biomembranes.

[bib28] Hartzog PE, Cain BD (1993). Mutagenic analysis of the a subunit of the F1F0 ATP synthase in Escherichia coli: Gln-252 through Tyr-263. Journal of Bacteriology.

[bib29] Hatch LP, Cox GB, Howitt SM (1995). The essential arginine residue at position 210 in the alpha subunit of the Escherichia coli ATP synthase can be transferred to position 252 with partial retention of activity. Journal of Biological Chemistry.

[bib30] Howitt SM, Lightowlers RN, Gibson F, Cox GB (1990). Mutational analysis of the function of the a-subunit of the F0F1-APPase of Escherichia coli. Biochimica Et Biophysica Acta (BBA) - Bioenergetics.

[bib31] Ishmukhametov RR, Pond JB, Al-Huqail A, Galkin MA, Vik SB (2008). ATP synthesis without R210 of subunit a in the Escherichia coli ATP synthase. Biochimica et biophysica acta.

[bib32] Jaenicke R, Böhm G (1998). The stability of proteins in extreme environments. Current Opinion in Structural Biology.

[bib33] Junge W, Lill H, Engelbrecht S (1997). ATP synthase: an electrochemical transducer with rotatory mechanics. Trends in Biochemical Sciences.

[bib34] Kato Y, Matsui T, Tanaka N, Muneyuki E, Hisabori T, Yoshida M (1997). Thermophilic F1-ATPase is activated without dissociation of an endogenous inhibitor, epsilon subunit. The Journal of Biological Chemistry.

[bib35] Klionsky DJ, Brusilow WS, Simoni RD (1984). In vivo evidence for the role of the epsilon subunit as an inhibitor of the proton-translocating ATPase of Escherichia coli. Journal of Bacteriology.

[bib36] Klusch N, Murphy BJ, Mills DJ, Yildiz Ö, Kühlbrandt W (2017). Structural basis of proton translocation and force generation in mitochondrial ATP synthase. eLife.

[bib37] Krah A, Pogoryelov D, Langer JD, Bond PJ, Meier T, Faraldo-Gómez JD (2010). Structural and energetic basis for H+ versus na+ binding selectivity in ATP synthase fo rotors. Biochimica Et Biophysica Acta (BBA) - Bioenergetics.

[bib38] Kumar S, Nussinov R (2001). How do thermophilic proteins deal with heat?. Cellular and Molecular Life Sciences.

[bib39] Laget PP, Smith JB (1979). Inhibitory properties of endogenous subunit epsilon in the Escherichia coli F1 ATPase. Archives of Biochemistry and Biophysics.

[bib40] Leone V, Pogoryelov D, Meier T, Faraldo-Gómez JD (2015). On the principle of ion selectivity in Na^+^/H^+^-coupled membrane proteins: experimental and theoretical studies of an ATP synthase rotor. PNAS.

[bib41] Lightowlers RN, Howitt SM, Hatch L, Gibson F, Cox GB (1987). The proton pore in the Escherichia coli F0F1-ATPase: a requirement for arginine at position 210 of the a-subunit. Biochimica Et Biophysica Acta (BBA) - Bioenergetics.

[bib42] Lightowlers RN, Howitt SM, Hatch L, Gibson F, Cox G (1988). The proton pore in the Escherichia coli F0F1-ATPase: substitution of glutamate by glutamine at position 219 of the alpha-subunit prevents F0-mediated proton permeability. Biochimica Et Biophysica Acta (BBA) - Bioenergetics.

[bib43] Marr CR, Benlekbir S, Rubinstein JL (2014). Fabrication of carbon films with ∼ 500nm holes for cryo-EM with a direct detector device. Journal of Structural Biology.

[bib44] Mayer F, Leone V, Langer JD, Faraldo-Gómez JD, Müller V (2012). A c subunit with four transmembrane helices and one ion (Na+)-binding site in an archaeal ATP synthase: implications for c ring function and structure. The Journal of Biological Chemistry.

[bib45] Mazhab-Jafari MT, Rohou A, Schmidt C, Bueler SA, Benlekbir S, Robinson CV, Rubinstein JL (2016). Atomic model for the membrane-embedded V_O_ motor of a eukaryotic V-ATPase. Nature.

[bib46] Meyerson JR, Rao P, Kumar J, Chittori S, Banerjee S, Pierson J, Mayer ML, Subramaniam S (2014). Self-assembled monolayers improve protein distribution on holey carbon cryo-EM supports. Scientific Reports.

[bib47] Mitome N, Suzuki T, Hayashi S, Yoshida M (2004). Thermophilic ATP synthase has a decamer c-ring: indication of noninteger 10:3 H+/ATP ratio and permissive elastic coupling. PNAS.

[bib48] Moore KJ, Angevine CM, Vincent OD, Schwem BE, Fillingame RH (2008). The cytoplasmic loops of subunit a of Escherichia coli ATP synthase may participate in the proton translocating mechanism. The Journal of Biological Chemistry.

[bib49] Morales-Rios E, Montgomery MG, Leslie AG, Walker JE (2015). Structure of ATP synthase from *Paracoccus denitrificans* determined by X-ray crystallography at 4.0 Å resolution. PNAS.

[bib50] Nalin CM, Cross RL (1982). Adenine nucleotide binding sites on beef heart F1-ATPase. Specificity of cooperative interactions between catalytic sites. The Journal of Biological Chemistry.

[bib51] Okazaki K, Hummer G (2015). Elasticity, friction, and pathway of γ-subunit rotation in FoF1-ATP synthase. PNAS.

[bib52] Pänke O, Cherepanov DA, Gumbiowski K, Engelbrecht S, Junge W (2001). Viscoelastic dynamics of actin filaments coupled to rotary F-ATPase: angular torque profile of the enzyme. Biophysical Journal.

[bib53] Paumard P, Vaillier J, Coulary B, Schaeffer J, Soubannier V, Mueller DM, Brèthes D, di Rago JP, Velours J (2002). The ATP synthase is involved in generating mitochondrial cristae morphology. The EMBO Journal.

[bib54] Pettersen EF, Goddard TD, Huang CC, Couch GS, Greenblatt DM, Meng EC, Ferrin TE (2004). UCSF chimera--a visualization system for exploratory research and analysis. Journal of Computational Chemistry.

[bib55] Pogoryelov D, Yildiz O, Faraldo-Gómez JD, Meier T (2009). High-resolution structure of the rotor ring of a proton-dependent ATP synthase. Nature Structural & Molecular Biology.

[bib56] Porter AC, Kumamoto C, Aldape K, Simoni RD (1985). Role of the b subunit of the Escherichia coli proton-translocating ATPase. A mutagenic analysis. The Journal of Biological Chemistry.

[bib57] Preiss L, Yildiz O, Hicks DB, Krulwich TA, Meier T (2010). A new type of proton coordination in an F(1)F(o)-ATP synthase rotor ring. PLoS Biology.

[bib58] Preiss L, Klyszejko AL, Hicks DB, Liu J, Fackelmayer OJ, Yildiz Ö, Krulwich TA, Meier T (2013). The c-ring stoichiometry of ATP synthase is adapted to cell physiological requirements of alkaliphilic Bacillus pseudofirmus OF4. PNAS.

[bib59] Preiss L, Langer JD, Yildiz Ö, Eckhardt-Strelau L, Guillemont JE, Koul A, Meier T (2015). Structure of the mycobacterial ATP synthase fo rotor ring in complex with the anti-TB drug bedaquiline. Science Advances.

[bib60] Punjani A, Rubinstein JL, Fleet DJ, Brubaker MA (2017). cryoSPARC: algorithms for rapid unsupervised cryo-EM structure determination. Nature Methods.

[bib61] Rees DM, Leslie AG, Walker JE (2009). The structure of the membrane extrinsic region of bovine ATP synthase. PNAS.

[bib62] Robert X, Gouet P (2014). Deciphering key features in protein structures with the new ENDscript server. Nucleic Acids Research.

[bib63] Rohou A, Grigorieff N (2015). CTFFIND4: fast and accurate defocus estimation from electron micrographs. Journal of Structural Biology.

[bib64] Rubinstein JL, Brubaker MA (2015). Alignment of cryo-EM movies of individual particles by optimization of image translations. Journal of Structural Biology.

[bib65] Russo CJ, Passmore LA (2014). Ultrastable gold substrates for electron cryomicroscopy. Science.

[bib66] Saita E, Iino R, Suzuki T, Feniouk BA, Kinosita K, Yoshida M (2010). Activation and stiffness of the inhibited states of F1-ATPase probed by single-molecule manipulation. Journal of Biological Chemistry.

[bib67] Schlegel K, Leone V, Faraldo-Gómez JD, Müller V (2012). Promiscuous archaeal ATP synthase concurrently coupled to Na+ and H+ translocation. PNAS.

[bib68] Sekiya M, Hosokawa H, Nakanishi-Matsui M, Al-Shawi MK, Nakamoto RK, Futai M (2010). Single molecule behavior of inhibited and active states of Escherichia coli ATP synthase F1 rotation. The Journal of Biological Chemistry.

[bib69] Shirakihara Y, Shiratori A, Tanikawa H, Nakasako M, Yoshida M, Suzuki T (2015). Structure of a thermophilic F1-ATPase inhibited by an ε-subunit: deeper insight into the ε-inhibition mechanism. FEBS Journal.

[bib70] Sielaff H, Rennekamp H, Wächter A, Xie H, Hilbers F, Feldbauer K, Dunn SD, Engelbrecht S, Junge W (2008). Domain compliance and elastic power transmission in rotary F(O)F(1)-ATPase. PNAS.

[bib71] Sobti M, Smits C, Wong ASW, Ishmukhametov R, Stock D, Sandin S, Stewart AG (2016). Cryo-EM structures of the autoinhibited *E. coli* ATP synthase in three rotational states. eLife.

[bib72] Soga N, Kinosita K, Yoshida M, Suzuki T (2012). Kinetic equivalence of transmembrane pH and electrical potential differences in ATP synthesis. Journal of Biological Chemistry.

[bib73] Srivastava AP, Luo M, Zhou W, Symersky J, Bai D, Chambers MG, Faraldo-Gómez JD, Liao M, Mueller DM (2018). High-resolution cryo-EM analysis of the yeast ATP synthase in a lipid membrane. Science.

[bib74] Stock D, Leslie AG, Walker JE (1999). Molecular architecture of the rotary motor in ATP synthase. Science.

[bib75] Stocker A, Keis S, Vonck J, Cook GM, Dimroth P (2007). The structural basis for unidirectional rotation of thermoalkaliphilic F1-ATPase. Structure.

[bib76] Suzuki T, Ueno H, Mitome N, Suzuki J, Yoshida M (2002). F(0) of ATP synthase is a rotary proton channel. obligatory coupling of proton translocation with rotation of c-subunit ring. The Journal of Biological Chemistry.

[bib77] Suzuki T, Murakami T, Iino R, Suzuki J, Ono S, Shirakihara Y, Yoshida M (2003). F0F1-ATPase/synthase is geared to the synthesis mode by conformational rearrangement of epsilon subunit in response to proton motive force and ADP/ATP balance. The Journal of Biological Chemistry.

[bib78] Szilágyi A, Závodszky P (2000). Structural differences between mesophilic, moderately thermophilic and extremely thermophilic protein subunits: results of a comprehensive survey. Structure.

[bib79] Tivol WF, Briegel A, Jensen GJ (2008). An improved cryogen for plunge freezing. Microscopy and Microanalysis.

[bib80] Trabuco LG, Villa E, Mitra K, Frank J, Schulten K (2008). Flexible fitting of atomic structures into electron microscopy maps using molecular dynamics. Structure.

[bib81] Vik SB, Cain BD, Chun KT, Simoni RD (1988). Mutagenesis of the alpha subunit of the F1Fo-ATPase from Escherichia coli. Mutations at Glu-196, Pro-190, and Ser-199.. The Journal of Biological Chemistry.

[bib82] Vik SB, Long JC, Wada T, Zhang D (2000). A model for the structure of subunit a of the Escherichia coli ATP synthase and its role in proton translocation. Biochimica Et Biophysica Acta (BBA) - Bioenergetics.

[bib83] Vik SB, Antonio BJ (1994). A mechanism of proton translocation by F1F0 ATP synthases suggested by double mutants of the a subunit. The Journal of Biological Chemistry.

[bib84] Vinothkumar KR, Montgomery MG, Liu S, Walker JE (2016). Structure of the mitochondrial ATP synthase from *Pichia angusta* determined by electron cryo-microscopy. PNAS.

[bib85] Wächter A, Bi Y, Dunn SD, Cain BD, Sielaff H, Wintermann F, Engelbrecht S, Junge W (2011). Two rotary motors in F-ATP synthase are elastically coupled by a flexible rotor and a stiff stator stalk. PNAS.

[bib86] Walker JE (2013). The ATP synthase: the understood, the uncertain and the unknown. Biochemical Society Transactions.

[bib87] Wang H, Oster G (1998). Energy transduction in the F1 motor of ATP synthase. Nature.

[bib88] Watt IN, Montgomery MG, Runswick MJ, Leslie AG, Walker JE (2010). Bioenergetic cost of making an adenosine triphosphate molecule in animal mitochondria. PNAS.

[bib89] Zhao J, Benlekbir S, Rubinstein JL (2015). Electron cryomicroscopy observation of rotational states in a eukaryotic V-ATPase. Nature.

[bib90] Zhou A, Rohou A, Schep DG, Bason JV, Montgomery MG, Walker JE, Grigorieff N, Rubinstein JL (2015). Structure and conformational states of the bovine mitochondrial ATP synthase by cryo-EM. eLife.

