# Supplementary material for: Structure of a bacterial ATP synthase
Source: eLife. 2019 Feb 6;8:e43128. doi: 10.7554/eLife.43128 (PMC6377231; doi:10.7554/eLife.43128)
Supplement: Supplementary file 1. — (A) Cryo-EM data acquisition and image processing. (B) Map and model statistics. (C) Residues included in atomic models. (D) Deposited maps and associated coordinate files. [file elife-43128-supp1.docx]

**Table 1.** Cryo-EM data acquisition, processing, atomic model statistics, and map/model depositions.

A. Cryo-EM data acquisition and image processing.

| **Data Collection** | |
| --- | --- |
| Electron Microscope | Titan Krios |
| Camera | Falcon 3EC |
| Voltage (kV) | 300 |
| Nominal Magnification | 75,000 |
| Calibrated physical pixel size (Å) | 1.06 |
| Total exposure (e/Å^2^) | 42.7 |
| Exposure rate (e/pixel/s) | 0.8 |
| Number of frames | 30 |
| Defocus range (μm) | 0.5 to 3.8 |
| **Image Processing** | |
| Motion correction software | *cryoSPARC v2* |
| CTF estimation software | *CTFFIND 4* |
| Particle selection software | *Relion 2.1* |
| Micrographs used | 10,940 |
| Particle images selected | 1,866,804 |
| 3D map classification and refinement software | *cryoSPARC v2, Relion 2.1* |

B. Map and model statistics.

| **EM maps** | Intact class 1 | Intact class 2 | | Intact class 3 | Focused F_O_/stalk class 1 | | Focused F_O_/stalk class 2 | Focused F_O_/stalk class 3 | | Focused F_O_ |
| --- | --- | --- | --- | --- | --- | --- | --- | --- | --- | --- |
| Particle images contributing to maps | 405,432 | 314,448 | | 175,694 | 405,432 | | 314,448 | 175,694 | | 895,574 |
| Applied symmetry | C1 | C1 | | C1 | C1 | | C1 | C1 | | C1 |
| Applied B-factor (Å^2^) | -96.5 | -93.1 | | -96.6 | -169.2 | | -153.1 | -186.3 | | -155.2 |
| Global resolution (FSC = 0.143, Å) | 3.0 | 3.0 | | 3.2 | 3.9 | | 3.9 | 4.8 | | 3.3 |
| **Model Building** | F_1_ class 1 | | F_1_ class 2 | | | F_1_ class 3 | | | F_O_ | |
| Modeling software | Coot, Phenix | | | | | | | | | |
| Residue numbers | 3492 | | 3488 | | | 3490 | | | 993 | |
| RMS bond length (Å) | 0.006 | | 0.007 | | | 0.009 | | | 0.0084 | |
| RMS bond angle (º) | 0.964 | | 1.010 | | | 1.088 | | | 1.48 | |
| Ramachandaran outliers (%) | 0.17 | | 0.12 | | | 0.14 | | | 0.00 | |
| Ramachandran favoured (%) | 96.03 | | 95.73 | | | 95.13 | | | 97.40 | |
| Clashscore | 6.13 | | 6.34 | | | 6.25 | | | 14.25 | |
| MolProbity score | 1.61 | | 1.64 | | | 1.68 | | | 1.78 | |
| EMRinger score | 4.50 | | 4.44 | | | 4.46 | | | 1.68 | |
| Ligand | 1 Pi  1 Mg-ADP  3 Mg-ATP | | 1 Pi  1 Mg-ADP  3 Mg-ATP | | | 1 Pi  1 Mg-ADP  3 Mg-ATP | | |  | |

C. Residues included in atomic models.

| **F_1_ subunits** | Class 1 | Class 2 | Class 3 |
| --- | --- | --- | --- |
| α_E_ | 2-501 | 3-501 | 8-501 |
| α_DP_ | 8-501 | 2-501 | 3-501 |
| α_TP_ | 2-501 | 8-501 | 2-501 |
| β_E_ | 1-470 | 1-469 | 1-469 |
| β_DP_ | 2-471 | 2-471 | 2-471 |
| β_TP_ | 1-471 | 1-471 | 1-471 |
| γ | 2-284 | 2-285 | 2-285 |
| δ | 2-176 | 2-176 | 2-176 |
| ε | 4-132 | 2-53, 58-131 | 4-131 |

| **F_O_ subunits** | Atomic model | Poly alanine |
| --- | --- | --- |
| a | 6-131, 152-191, 198-234 |  |
| b_1_ | 7-49 | 50-163 |
| b_2_ | 7-43 | 44-140, C-terminal α-helix 600-616 |
| c_0_ – c_9_ | 2-72 |  |

D. Deposited maps and associated coordinate files.

| **Maps** | **EMDB code** | **Associated PDB ID** |
| --- | --- | --- |
| Intact class 1 | EMD-9333 | 6N2Y |
| Intact class 2 | EMD-9334 | 6N2Z |
| Intact class 3 | EMD-9335 | 6N30 |
| Focused F_O_/stalk class 1 | EMD-9336 |  |
| Focused F_O_/stalk class 2 | EMD-9337 |  |
| Focused F_O_/stalk class 3 | EMD-9338 |  |
| Focused F_O_ | EMD-9327 | 6N2D |
